# Hydrogen peroxide and extracellular signal-related kinase 1/2 pathway regulate ferritin levels in retinal pigmented and lens epithelial cells

**Published:** 2013-10-04

**Authors:** Marilyn M. Lall, Jill Harned, M. Christine McGahan

**Affiliations:** Department of Molecular Biomedical Sciences, North Carolina State University, Raleigh, NC

## Abstract

**Purpose:**

Iron plays a central role in the oxidative stress caused by hydrogen peroxide. The ubiquitous iron storage protein, ferritin, safely sequesters iron, reducing its ability to cause oxidative damage. Oxidative stress can activate mitogen-activated protein (MAP) kinase pathways with many downstream effects. The purpose of this study was to determine the effects of hydrogen peroxide on MAP kinase pathways (extracellular signal-related kinase [ERK]1/2, c-Jun N-terminal kinase [JNK], and p38) and ferritin levels in canine lens and retinal epithelial cells (lens epithelial cells [LECs] and retinal pigmented epithelial [RPE] cells).

**Methods:**

Primary cultures of canine LECs and RPE cells were used in these studies. Hydrogen peroxide was delivered either by a single 250 μM bolus or 0.25 mU/ml glucose oxidase (GO). Immunoblotting was used to determine the activation of the MAP kinase pathways. Ferritin was detected with enzyme immunosorbent assay.

**Results:**

Baseline activation of ERK1/2 in the untreated RPE cells and LECs was decreased by treatment with U-0126. Bolus hydrogen peroxide greatly increased ERK1/2 activation that had been blocked by U-0126, whereas GO had no significant effect on ERK1/2 phosphorylation. Hydrogen peroxide, either bolus or constant low levels, increased ferritin levels in the LECs and RPE cells. Surprisingly, U-0126 not only did not inhibit the effect of hydrogen peroxide on the ferritin levels but also increased the ferritin levels in both cell types. Neither bolus nor chronic hydrogen peroxide exposure activated the JNK or p38 pathway. Additionally, neither JNK nor p38 inhibitors had any effect on the ferritin concentrations in the LECs or RPE cells.

**Conclusions:**

Although U-0126 inhibited the hydrogen peroxide–induced increase in ERK1/2 phosphorylation, U-0126’s lack of inhibition of the peroxide-induced increase in intracellular ferritin levels indicates that this pathway is not involved in ferritin induction by hydrogen peroxide. This is the first study to demonstrate that hydrogen peroxide and an inhibitor of ERK1/2 activation can increase the levels of the iron storage protein, ferritin. Since ferritin can shield cells from iron-catalyzed damage, this downstream effect likely plays a protective role, which, in the case of the ERK1/2 inhibitor, U-0126, demonstrates a potential therapeutic target.

## Introduction

Mitogen-activated protein kinases (MAPKs) play an important role in cellular signaling in response to numerous stimuli including growth factors and oxidative stress [1]. Three MAPK sub-families have been defined: p38 kinase, c-Jun N-terminal kinase (JNK), and extracellular signal-related kinase (ERK). When activated by phosphorylation, they in turn phosphorylate specific substrates that activate various transcription factors. Hydrogen peroxide (H_2_O_2_) generated during cellular metabolism or released by activated leukocytes is an important contributor to oxidative stress. The cellular response to H_2_O_2_ has been studied intensively, but many aspects of this response remain unclear.

Adding H_2_O_2_ causes cell-type specific downstream effects, including the differential activation of members of the MAPK family. In osteoblasts, H_2_O_2_ caused sustained activation of ERK, but only transient (<30 min) activation of p38 and JNK. H_2_O_2_ caused cell death that was inhibited by blocking ERK activation, but not the inhibition of JNK or p38 activation [2,3]. Selenium protected osteoblasts against H_2_O_2_ damage by inhibiting ERK activation [4]. In contrast, cardiomyoblasts treated with H_2_O_2_ increased the phosphorylation of all three MAPK subfamilies (JNK, p38, and ERK1/2). These cells had increased heme oxygenase I (HO-1) messenger RNA (mRNA) and protein levels in response to H_2_O_2_. However, only the JNK and p38 pathways are apparently involved in this activation since ERK1/2 inhibition failed to block this effect [5]. Subtoxic levels of H_2_O_2_ increased production of the proinflammatory cytokine, interleukin-6, in cultured human retinal pigment epithelial (RPE) cells. This effect was solely due to the activation of p38 kinase [6].

Interestingly, the hemoglobin degradation product, hemin, induces HO-1 (which can catabolize hemin and cause oxidative stress) in mixed neuron-astrocyte cultures and causes neurotoxicity. In this case, inhibition of ERK1/2, but not the JNK and p38 pathways, decreases HO-1 expression and toxicity caused by hemin addition. In another study, the ERK1/2 inhibitor U-0126 decreased HO-1 activity as well as hemoglobin neurotoxicity in murine cortical cell cultures [7]. The investigators concluded that ERK1/2 inhibitors appear to act as antioxidants.

Iron is central to the damaging effects of H_2_O_2_ because iron participates in the in vivo superoxide-driven Fenton chemistry [8]. Normally, iron is safely bound to enzymes of which it is an essential part, or is stored inertly in the ubiquitous iron storage protein, ferritin [9]. Iron storage in ferritin protects cells from oxidative damage. Indeed, the central role that iron plays in H_2_O_2_ damage of RPE cells is clearly demonstrated by the complete inhibition of peroxide-induced damage to these cells by an iron chelator [10]. Additionally, iron-induced damage is strongly implicated in the pathophysiology of cataractogenesis and numerous neurologic and retinal degenerations, including age-related macular degeneration (AMD) [10-14]. Iron-induced oxidative stress is likely responsible for the activation of MAPK. The cellular content of ferritin responds to oxidative stress. Because of previous compelling studies [10-14] linking iron to oxidative stress and damage in ocular tissues, the purpose of the current study was to determine whether H_2_O_2_ activates MAPK pathways resulting in alteration in the levels of the protective iron storage protein ferritin in primary cultures of lens epithelial cells (LECs) and RPE cells. To our knowledge, there have been no previous studies on oxidative stress, MAPK, and ferritin metabolism in any cell type.

## Methods

### Tissue culture

Dogs were obtained from Johnston County Animal Shelter, NC, after they had been euthanized. The eyes were removed from the orbits within 3 h of death and cut 7–8 mm posterior to the limbus. The lens was dissected free from the anterior portion of the globe. The anterior lens capsule with adherent epithelial cells was removed from the lens and placed in 10 cm tissue culture plates containing Dulbecco’s Modified Eagle Medium (Invitrogen, Rockville, MD) supplemented with 10% fetal bovine serum (Hyclone, Logan, UT) and 1% antibiotic antimycotic solution (Mediatech, Herndon, VA) at 37 °C in a humidified environment containing 95% air and 5% CO_2_. After significant outgrowth of epithelial cells from the capsule, the cells were dispersed and grown to confluence. They were then seeded into six-well plates for experiments. Treatments were performed on 80–90% confluent cultures with serum-free, L-glutamine-free minimum essential medium (MEM; Invitrogen) unless otherwise noted.

To culture RPE cells, the posterior section of the globe was placed in a cup-shaped dissection dish to maintain the normal shape of the globe. After the vitreous and the retina were removed, Hanks’ balanced salt solution without calcium or magnesium (Mediatech, Manassas, VA) and supplemented with 0.05% Trypsin and 0.53 mM EDTA was added to the exposed RPE cells. After incubation for 10 min at 37 °C, patches of RPE cells were lifted and transferred to a centrifuge tube containing Ham’s F12-Dulbecco’s Modified Eagle Medium (1:1, Invitrogen) supplemented with 20% fetal bovine serum (Hyclone) and 1% antibiotic antimycotic solution (Mediatech) and spun at 150 ×g for 5 min. Cells were then plated in 60 mm dishes and incubated at 37 °C in 95% air, 5% CO_2_, and 95% humidity. The cultures became confluent after 6−10 days. The RPE cells were then seeded into six-well plates for the experiments. Treatments were performed on 80–90% confluent cultures with serum-free, L-glutamine-free MEM (Invitrogen) unless otherwise noted.

### Materials

1,4-Diamino-2,3-dicyano-1,4-bis[2-aminophenylthio]butadiene (U-0126), 4-(4-Fluorophenyl)-2-(4-methylsulfinylphenyl)-5-(4-pyridyl)1H-imidazole (SB203580, SB), and Anthra[1,9-cd]pyrazol-6(2H)-one (SP600125, SP) were purchased from Biomol (Plymouth Meeting, PA). H_2_O_2_, glucose oxidase (GO), horseradish peroxidase type II (HRP), and o-dianisidine dihydrochloride (o-DD) were obtained from Sigma (St. Louis, MO). 2,2’dipyridyl-N,N-dimethylsemicarbazone (Dp) was obtained from Calbiochem (Billerica, MA).

### Measurement of hydrogen peroxide

H_2_O_2_ generated from GO was measured according to a method previously described [15]. Briefly, 14.2 U/ml HRP and 0.08 mg/ml o-DD were added to a 96-well plate containing 0.25 mU/ml glucose oxidase. The plate was incubated at 37 °C for 1 h, and absorbance measured at 470 nm on a microplate reader (Tecan, Research Triangle park, NC).

### Cell proliferation

Seventy to ninety percent confluent LECs were exposed to U-0126 for 10 min followed by [3H]Thymidine for 24 h. The cells were then washed twice with ice-cold PBS (Corning Cellgro without calcium and magnesium, Cat #21-040-CV containing sodium chloride and sodium phosphate, Mediatech). Five percent tricholoracetic acid (TCA) was added to each well and allowed to incubate on ice for 10 min for a total of two washes. Next, the LECs were rinsed with ice-cold PBS three times. Then 0.2 N NaOH was added, and shaken on ice for 30 min. The cells were scraped, and 478 μl lysate was transferred to a vial containing 10 ml scintillation fluid (National Diagnostics, Atlanta, GA), and radioactivity was measured using a 1409 Wallac liquid scintillation counter (Perkin Elmer, Wellesley, MA). The remaining lysate was used to determine the total amount of protein.

### Immunoblotting for identifying extracellular signal-related kinase 1/2 protein

Cytosolic extracts, prepared from primary cultured canine LECs, were analyzed with immunoblotting for ERK1/2. Nerve growth factor–treated PC12 (rat adrenal medulla pheochromocytoma) cell extracts were used as positive control (Promega, Madison, WI) and untreated PC12 cell extracts as negative control (Promega). Activation of MAPK signaling pathways results in phosphorylation of ERK1/2. Ten micrograms of total protein from primary cultured canine LECs and 2 μg nerve growth factor–treated and untreated PC12 cell extracts were subjected to 10% sodium dodecyl sulfate–polyacrylamide gel electrophoresis. After electrophoresis, the proteins were transferred to a nitrocellulose membrane. The membrane was blocked with 1% bovine serum albumin in Tris-buffered saline (TBS) and then probed with antibodies against ERK1/2 and its phosphorylated forms (1:5,000; Promega). After being washed with Tris-buffered saline containing Tween-20 (TBST), the membrane was incubated with horseradish peroxidase–conjugated donkey antirabbit immunoglobulin G antibodies (1:5,000; Promega). Proteins were visualized using the enhanced chemiluminescence detection system (Amersham, Piscataway, NJ) according to the manufacturer’s protocol.

### Ferritin detection with enzyme-linked immunosorbent assay

Total ferritin concentration was measured in whole cell lysates with a sandwich enzyme-linked immunosorbent assay. Plates are coated overnight with 4 μg/ml purified goat antihorse ferritin (Bethyl Laboratories, Montgomery, TX) and then blocked with 1% gelatin in PBS for 1 h. Standards (dog liver ferritin) and samples were added to the wells and incubated for 1 h at 37 °C. After three washes with 0.05% Tween-20 in PBS, 1 μg/ml horseradish peroxidase–labeled goat antihorse ferritin (Bethyl) was added to each well. Following 1 h incubation at room temperature, the plate was washed five times. 2,29-azino-di[3-ethyl-benzthiazolinesulfonate] (ABTS) [6] substrate (KPL Labs, Gaithersburg, MD) was added to each well, and the plate was incubated for 20 min at room temperature. After the color developed (30 min), the plate was read at 450 nm using a microplate reader (Tecan).

### Statistical analysis

Results are presented as the mean±standard error of the mean (SEM). Statistical analysis of the data was performed with Systat, version 9.01 (San Jose, CA). An unpaired Student *t* test was used to determine the statistical significance between two group means. When analysis involved more than two means, one-way analysis of variance (ANOVA) was conducted, and the significance of the differences between the means was determined using Tukey’s test. The difference in means is considered significant when p<0.05.

## Results

### Hydrogen peroxide treatment of lens epithelial cells

**Figure 1:**
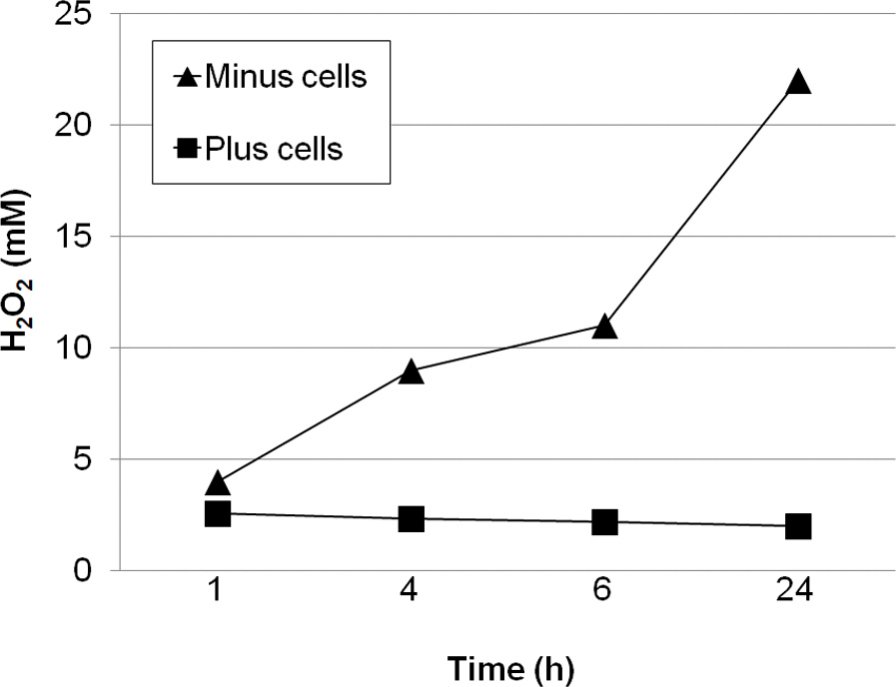
Generation of hydrogen peroxide by glucose oxidase. Time course for the production of hydrogen peroxide (H_2_O_2_) by 0.25 mU/ml glucose oxidase (GO) in the presence and absence of lens epithelial cells.

H_2_O_2_ was delivered to LECs by either a single 250 μM bolus or glucose oxidase (GO), which produces a continuous supply of H_2_O_2_ to the medium. As shown by others, a bolus of H_2_O_2_ quickly disappeared from the medium (data not shown) due to the highly active antioxidant defense present in these cells [16]. Testing a series of different amounts of GO, we determined that adding 0.25 mU/ml GO to LECs resulted in a steady-state level of H_2_O_2_ of approximately 2.0 μM over a 24 h period (Figure 1). When cells were not present, H_2_O_2_ continued to accumulate in the medium until the H_2_O_2_ concentration reached more than 20 μM by 24 h, indicating that LECs contain a robust system for eliminating this oxidant.

### Extracellular signal-related kinase 1/2 inhibitor effects on hydrogen peroxide–induced phosphorylation of extracellular signal-related kinase 2 and cellular proliferation

**Figure 2:**
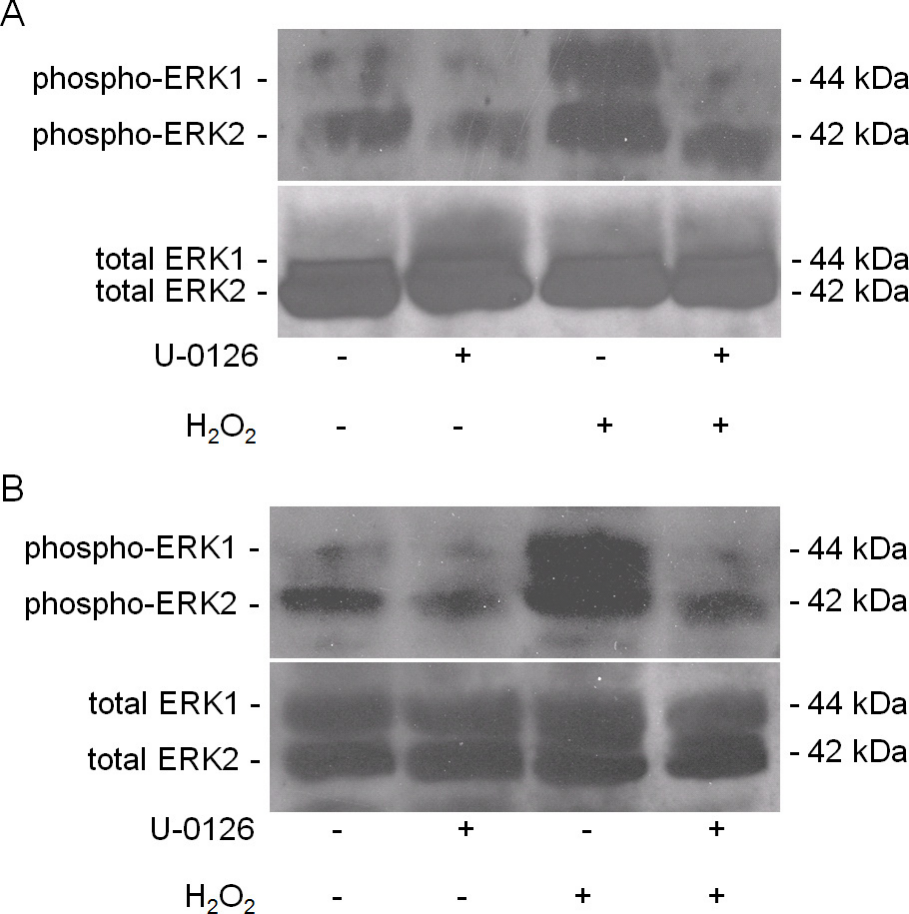
U-0126 blocks hydrogen peroxide–induced activation of extracellular signal-related kinase 1/2. Western blot of extracellular signal-related kinase 1/2 (ERK1/2) obtained from retinal pigment epithelial (RPE) cells was probed with antibody specific for ERK1/2 (42 and 44 kDa). Phospho-ERK1/2 expression (upper panel) and ERK1/2 (lower panel) in (**A**) lens epithelial cells (LECs) and (**B**) RPE cells treated with 250 μM H_2_O_2_ in the presence or absence of U-0126 for 24 h.

**Figure 3:**
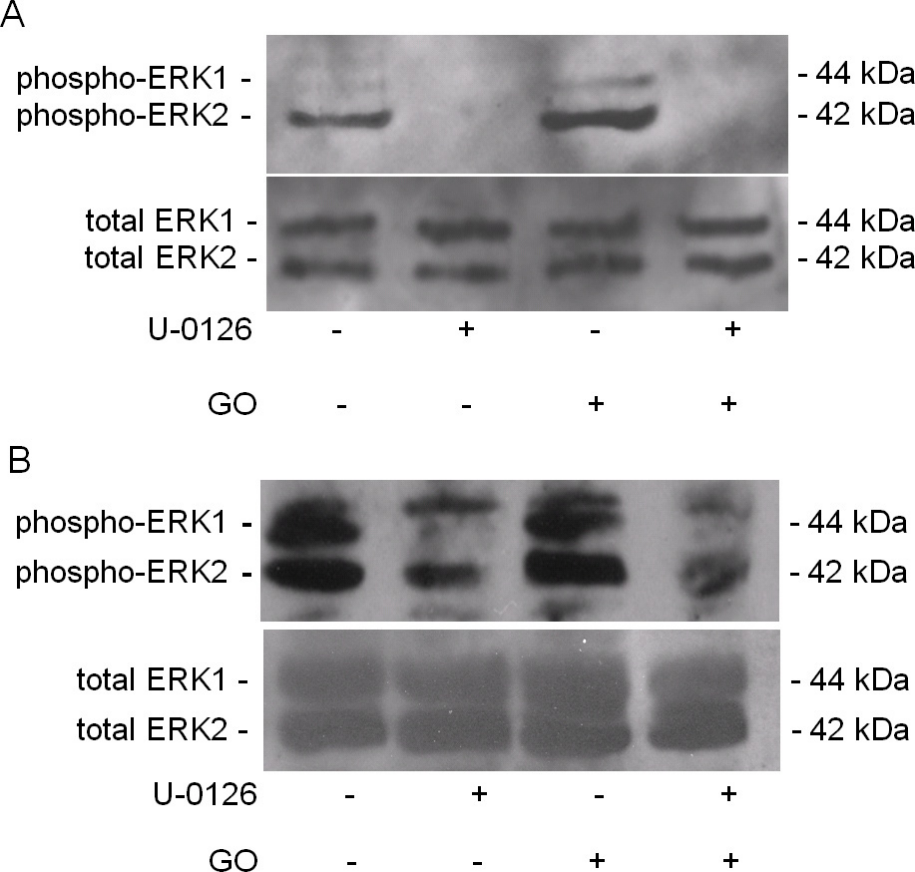
The effect of glucose oxidase on extracellular signal-related kinase 1/2 phosphorylation. Phosphoextracellular signal-related kinase 1/2 (ERK1/2) expression (upper panel) and total ERK1/2 (lower panel) in (**A**) lens epithelial cells (LECs) and (**B**) retinal pigment epithelial (RPE) cells treated with 0.25 mU/ml glucose oxidase in the presence or absence of 10 μM U-0126 for 24 h.

**Figure 4:**
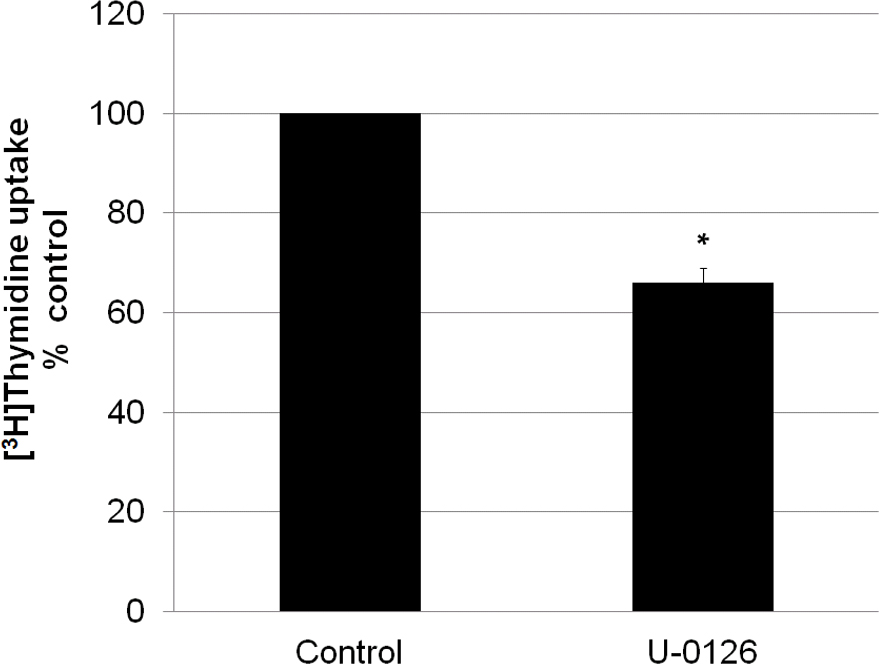
Inhibition of extracellular signal-related kinase 1/2 decreases lens epithelial cell proliferation. Lens epithelial cells (LECs) were exposed to 10 μM U-0126 for 10 min followed by the addition of 1 μCi/ml [3H]thymidine for 24 h. Each histogram bar represents the mean±SEM of at least six samples. *p=0, significantly different from control. Statistical tests for all data included ANOVA and Tukey’s test.

Activation of MAPK signaling pathways results in phosphorylation of ERK1/2. The control LECs and RPE contained some phosphorylated ERK1/2 that disappeared upon treatment with the ERK1/2 inhibitor, U-0126 (Figure 2). The increase in ERK1/2 phosphorylation with bolus H_2_O_2_ treatments in the LECs (Figure 2A) and RPE cells (Figure 2B) was significantly decreased by U-0126. The effect of GO on ERK1/2 phosphorylation in LECs (Figure 3A) and RPE cells (Figure 3B) was notably less than the effect of bolus H_2_O_2_. U-0126 also inhibited phosphorylation in this set of experiments. Interestingly, U-0126 caused a significant decrease in LEC proliferation as shown by a decrease in [3H]thymidine incorporation (Figure 4).

### Ferritin levels in lens epithelial cells and retinal pigment epithelium

**Figure 5:**
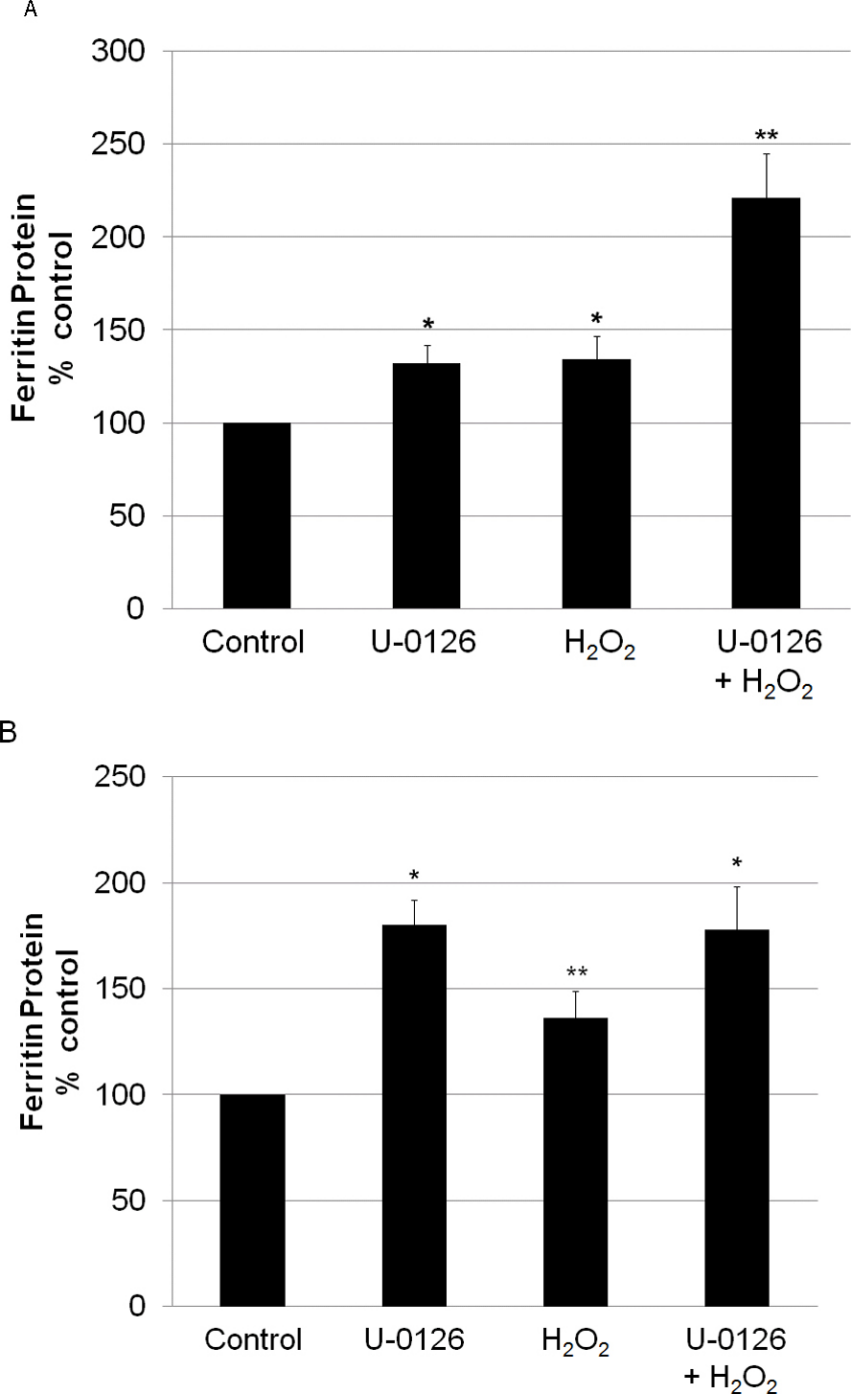
The effects of U-0126 and hydrogen peroxide on ferritin levels in lens epithelial cells and retinal pigment epithelial cells. (**A**) Lens epithelial cells (LECs) or (**B**) retinal pigment epithelial (RPE) cells were treated with either 10 μM U-0126 or 250 μM hydrogen peroxide (H_2_O_2_) or both for 20 h. Cells treated with both were pretreated for 10 min with U-0126 before H_2_O_2_ was added. Ferritin content of the cells was measured with enzyme-linked immunosorbent assay (ELISA). Each histogram bar represents the mean±SEM of at least six samples. **A**: *p=0, significantly different from control and U-0126, **p<0.05, significantly different from all other groups. **B**: *p<0.05, significantly different from control and H_2_O_2_. **p<0.05, significantly different from all other groups. Statistical tests for all data included ANOVA and Tukey’s test.

Adding a bolus of H_2_O_2_ resulted in a significant increase in the ferritin levels in the LECs. U-0126 did not block this increase but increased the ferritin levels in the LECs. Surprisingly, adding U-0126 to the LECs 10 min before H_2_O_2_ was added caused a significantly greater increase in the ferritin levels compared to either U-0126 or H_2_O_2_ alone (Figure 5A). Data from the RPE cells indicate that H_2_O_2_ increased the ferritin levels, which was not blocked by U-0126. In addition, as in the LECs, U-0126 alone caused an increase in ferritin levels. However, unlike in the LECs, it was not additive to the H_2_O_2_ effect (Figure 5B).

**Figure 6:**
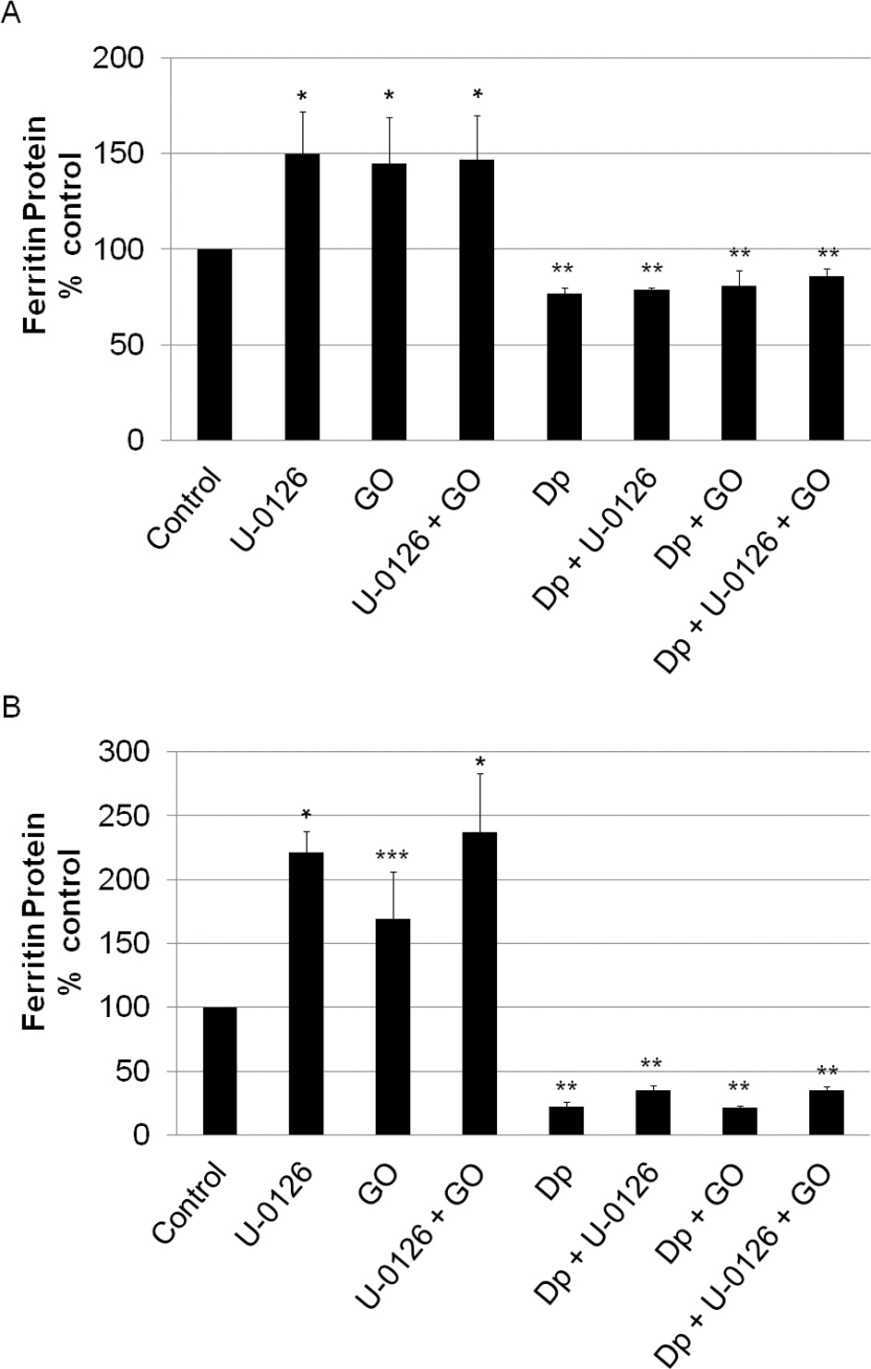
The effects the iron chelator 2,2’dipyridyl-N,N-dimethylsemicarbazone on U-0126 and glucose oxidase induced increases in ferritin levels in retinal pigment epithelial cells and lens epithelial cells. Retinal pigment epithelial (RPE) cells and lens epithelial cells (LECs) were treated with 10 μM U-0126, 0.25 mU/ml glucose oxidase (GO), and/or 0.3 mM 2,2’dipyridyl-N,N-dimethylsemicarbazone (Dp) for 24 h. U-0126-treated cells were pretreated with the extracellular signal-related kinase 1/2 (ERK1/2) inhibitor for 10 min before GO and/or Dp was added. Ferritin levels were measured with enzyme-linked immunosorbent assay (ELISA). Each histogram bar represents the mean±SEM of at least six samples. **A**: *p=0, significantly different from control. **p=0, significantly different from all groups without Dp added. **B**: *p=0, significantly different from control. **p=0, significantly different from all groups without Dp added. ***p=0, significantly different.

Steady-state production of low levels of H_2_O_2_ by GO for 24 h also resulted in an increase in ferritin levels in both cell types. As shown in Figures 5A,B, U-0126 also increased the ferritin concentrations in the LECs and the RPE cells (Figure 6A,B). In contrast to the additive effects of H_2_O_2_ and U-0126 on the ferritin concentrations in the LECs, GO and U-0126 had no additive effect on either cell type. To determine if the effects of U-0126 or GO on the ferritin concentrations were mediated by iron, the iron chelator Dp was added to the medium. Dp completely blocked the effects of U-0126, GO, and their combination in both cell types. Interestingly, iron chelation decreased the ferritin levels in the RPEs by 75% compared to 25% in the LECs. There is an additional difference between the RPE cells and the LECs as the increase in the ferritin levels in the LECs (140%–150% of control) was lower than that in the RPE cells (180%–240% of control) in response to U-0126 (Figure 5 and Figure 6).

The effect of inhibiting the other two MAPK pathways, JNK and p38, on the H_2_O_2_-induced increase in ferritin levels was also determined. Neither SP600125 (inhibitor of JNK) nor SB203580 (inhibitor of p38) had any effect on baseline ferritin levels, nor did the inhibitors inhibit the effects of H_2_O_2_ on this protein (data not shown).

## Discussion

The ability of LECs to catabolize H_2_O_2_ has been known for many years. Numerous studies have examined the toxic effect of this oxidant in many cell types and systems. However, the physiologic relevance of the amount of peroxide used in these studies has been controversial. Many studies have used large, non-physiologic levels of peroxide as high as 1 mM. In the present study, we compared the effects of a large bolus of H_2_O_2_ (250 μM) and a constant supply of a low level of H_2_O_2_ (2.0 μM) delivered by adding glucose oxidase or GO (0.25 mU/ml) on activation of MAPK pathways and ferritin levels in primary cultures of two different epithelial cell types, LECs and RPE cells. These cells actively consume H_2_O_2_ [16]. In addition, GO caused a linear increase in the peroxide concentration up to 40 μM in 24 h in the absence of cells, but in the presence of cells, consistent metabolism of the peroxide produced by GO resulted in a steady-state level of peroxide equal to 2 μM. LECs and RPE cells were chosen for this study because of the known contribution of oxidative damage to lenticular and retinal pathology and our limited understanding of the underlying mechanisms.

The controversy surrounding experimental design in studies of the effects of peroxide may find some resolution in the surprising results of the current study where a single large bolus of H_2_O_2_ had effects similar to those of constant low-level generation of H_2_O_2_ by GO on increasing ferritin levels in LECs and RPE cells. Our initial hypothesis was that the peroxide-induced increase in the ferritin concentration was caused by the activation of a MAPK pathway. We analyzed the activation of three pathways, JNK, p38, and ERK1/2, by H_2_O_2_ in LECs and RPE cells. Bolus addition of H_2_O_2_, but not chronic low-level peroxide generation by GO, caused a large increase in ERK1/2 phosphorylation. However, although U-0126, an inhibitor of ERK1/2 activation, blocked this activation by bolus addition of peroxide, U-0126 did not block the effects of peroxide on the increasing ferritin levels. Indeed, U-0126 itself not only caused an increase in ferritin concentration in the LECs and RPE cells but was also additive to the effects of bolus H_2_O_2_ on the ferritin concentration in the LECs (but not the RPE cells). This additivity was not seen when U-0126 was added with GO in either cell type. Therefore, activation of the ERK1/2 pathway by peroxide delivered either as a bolus or by chronic generation is not responsible for the increase in the ferritin concentration in these cells. Neither JNK nor p38 was activated by H_2_O_2_ in the LECs or the RPE cells, and inhibitors of these pathways did not alter either the baseline ferritin levels or the H_2_O_2_-induced increase in the ferritin concentration.

The effect of U-0126 on ferritin synthesis and concentration in LECs and RPE cells is intriguing and indicates that the ERK1/2 pathway is significantly involved in regulating ferritin synthesis. GO, bolus H_2_O_2_, and U-0126 all increased ferritin levels in the RPE cells and the LECs. These effects were all significantly blocked by an iron chelator, indicating that iron is central to these responses. The lack of additivity of H_2_O_2_ and U-0126 in all but the case of bolus H_2_O_2_ and U-0126 in LECs also points to a common mechanism of action.

Ferritin synthesis is acutely regulated at the translational level by iron [17]. It is difficult to explain the large difference in the effects of the iron chelator on the baseline and stimulated ferritin levels between the RPE cells (75% decrease) and the LECs (25% decrease). There might be different levels of iron accessible to chelators in these different cell types. It is also interesting that U-0126 caused a larger increase in ferritin levels in the RPE cells than the LECs. This might provide additional evidence that iron may be more accessible in RPE cells than in LECs for stimulating ferritin synthesis.

The U-0126-induced decrease in cell proliferation seen here in the LECs could also cause a decrease in the need for iron availability and an increased need for iron storage, and thus an increase in the ferritin levels in the cells. An increase in ferritin levels is generally thought to be protective against iron-induced oxidative damage since ferritin has such a large iron storage capacity. The H_2_O_2_-induced increase in ferritin levels that we determined could also explain the well-documented ability of LECs to adapt to stress caused by H_2_O_2_ exposure [18,19]. An increase in the levels of a protective protein resulting from U-0126 exposure matches well with other studies indicating that inhibition of ERK1/2 has protective effects in neurons against heme oxygenase, hemoglobin, and heme-induced injury [7,20]. However, in one of these studies, U-0126 inhibited the hemin-induced increase in ferritin expression. The effects of U-0126 alone on ferritin expression were not shown in that study. The differences in the effects of U-0126 on oxidative stress–induced increase in ferritin levels in the current study and that of Benvenisit-Zaroom could be due to the different stimulant (hemin) and/or cell type (mixed neuron-astrocyte cultures) used in the earlier study. To our knowledge, this is the first study to demonstrate that H_2_O_2_ and an inhibitor of ERK1/2 activation can independently increase the levels of the iron storage and protective protein ferritin in any cell type. Importantly, inhibition of the ERK1/2 pathway could provide a route to development of therapeutic agents for various diseases with an iron-linked oxidative stress component.
